# Evaluation of the Ocular Surface and Meibomian Gland in Obstructive Sleep Apnea Hypopnea Syndrome

**DOI:** 10.3389/fmed.2022.832954

**Published:** 2022-02-09

**Authors:** Shaohua Liu, Shisheng Li, Mengmeng Li, Shiying Zeng, Baihua Chen, Liwei Zhang

**Affiliations:** ^1^Department of Ophthalmology, The Second Xiangya Hospital of Central South University, Changsha, China; ^2^Hunan Clinical Research Centre of Ophthalmic Disease, Changsha, China; ^3^Department of Otorhinolaryngology, The Second Xiangya Hospital of Central South University, Changsha, China

**Keywords:** obstructive sleep apnea hypopnea syndrome, dry eye, ocular surface, meibomian gland, tear film

## Abstract

**Purpose:**

To assess the ocular surface and meibomian gland (MG) of patients with obstructive sleep apnea hypopnea syndrome (OSAHS) and to explore the effects of surgery for OSAHS on the ocular surface and MG.

**Methods:**

Based on the apnea hypopnea index (AHI), 21 patients with mild OSAHS (Group A, 5/h ≤ AHI < 15/h), 20 patients with moderate OSAHS (Group B, 15/h ≤ AHI < 30/h), 62 patients with severe OSAHS (Group C, AHI ≥ 30/h) were examined. The ocular surface and MG were evaluated using Keratograph 5M. In addition, detailed Ophthalmic examination including visual acuity, refraction, slit-lamp examination of the anterior segment, corneal fluorescein staining (CFS), ocular surface disease index (OSDI) scoring, Schirmer I test (SIT) and serum lipid measurement was performed. For OSAHS patients with dry eye syndrome (DES) who underwent uvulopalatopharyngoplasty for improving AHI, the conditions of the ocular surface and MG were compared before surgery and 3 months after surgery. Only the data of the right eyes were analyzed.

**Results:**

There were no significantly different in the OSDI score, tear meniscus height (TMH), or loss ratio of the lower eyelid (LRLE) among these groups. The first non-invasive tear film breakup time (fNIBUT), average non-invasive tear film breakup time (avNIBUT), bulbar redness index (BRI), lipid layer grading (LLG), CFS, plugged orifices and distortion in MG, the loss ratio of upper eyelid (LRUE), and the incidence of DES, floppy eyelid syndrome (FES) and meibomian gland dysfunction (MGD) showed significant differences between Groups A and C (*p* = 0.015, *p* = 0.018, *p* < 0.001, *p* = 0.022, *p* = 0.036, *p* = 0.007, *p* = 0.019, *p* = 0.017, *p* = 0.045, *p* = 0.013, and *p* = 0.029, respectively). The SIT in the Group A was significantly higher than in Group B (*p* = 0.025) and in Group C (*p* < 0.001). In the correlation analyses, the fNIBUT, avNIBUT, SIT and LLG had negative correlations with the AHI (*p* = 0.013, *p* = 0.010, *p* = 0.003, *p* < 0.001, and *p* = 0.006, respectively). The BRI, CFS and LRUE were positively correlated with the AHI (*p* = 0.006, *p* = 0.007, and *p* = 0.046, respectively). Three months after surgery, there were no significant differences in the ocular surface or MG.

**Conclusion:**

Patients with severe OSAHS have poor stability of tear film and are prone to lipid-deficient dry eye as a result of the loss of meibomian gland. By improving the AHI, the ocular surface damage of OSAHS patients cannot be reversed in a short time.

## Introduction

Obstructive sleep apnea hypopnea syndrome (OSAHS) is a chronic intermittent hypoxic sleep-disordered breathing disease that is characterized by repeated apnea and hypopnea during sleep (1). The prevalence of OSAHS is 2–4% in symptomatic patients and 24% in asymptomatic patients, especially in middle-aged overweight men (2, 3). Long-term intermittent hypoxia, hypercapnia, heightened sympathetic nervous system activity and increased catecholamine levels constitute the pathophysiological mechanism of OSAHS (4). The systemic hypertension, atheromatosis and autonomic nerve dysfunction caused by OSAHS may give rise to a series of complications, such as coronary atherosclerotic heart disease, diabetes mellitus, heart failure and even death (5, 6). Ocular blood vessels are also affected by these mechanisms, including increased oxidative stress, systemic inflammation and sympathetic activity (4). OSAHS is associated with various eye diseases, including glaucoma, optic neuropathy, optic papilledema and ocular surface disease (7–15). This association can be explained that long-term intermittent hypoxia increases the levels of carbon dioxide in blood and causes hemodynamic changes, such as large nocturnal fluctuations in blood pressure, dilation and increased volume of cerebral vessels. It disturbs the normal ocular hemodynamics, resulting in a series of ocular disorders eventually (16, 17). The ocular surface manifestations of OSAHS present as floppy eyelid syndrome (FES), papillary conjunctivitis, punctate epithelial keratopathy, keratitis and keratoconus (7–11).

Previous studies have found that OSAHS is associated with reduced tear film breakup time (TBUT) and Schirmer values, and increased ocular surface disease (OSDI) scores and corneal fluorescein staining (CFS), these associations show that ocular surface parameters are impaired in OSAHS and suggest a tendency toward dry eye syndrome (DES) in these patients (18, 19). Meibomian gland dysfunction (MGD) is a common etiology of DES (20), and there are only two researches that have explored the relationship between meibomian gland (MG) and OSAHS. It has been demonstrated that MG are lost in OSAHS, especially severe OSAHS (21, 22). In recent years, non-invasive examination has begun to be frequently applied for the evaluation of dry eye and MG (23–25). Keratograph 5M is a non-invasive corneal topographer that measures non-invasive breakup time by the irregular change of ring pattern projected by the Placido disc onto the surface of the tear film (26). Compared with invasive examination, Keratograph 5M does not need to instill fluorescein, which can avoid physical interference following invasive procedures, such as reflex tearing, drop size and concentration of fluorescein (26–28). It has great reproductivity and reliability in dry eye tests as a non-invasive and objective examination (29–31), and can observe distinct morphological changes in MG with infrared imaging technology. We have also included more observation indices, including tear meniscus height, lipid layer and bulbar redness index.

The treatment of OSAHS includes conservative approaches, such as weight loss, avoiding smoking, excessive alcohol and sedatives, continuous positive airway pressure (CPAP) and surgical therapy (32–34). Although CPAP has always been the preferred therapy for moderate to severe OSAHS (33), the effects of CPAP for improving AHI on the ocular surface are unclear. Kadyan et al. and Acar et al. have found that ocular surface damage improves along with OSAHS severity after continuous positive airway pressure (CPAP) therapy (35, 36). However, Hayiric et al. found that ocular surface damage was exacerbated after CPAP therapy, although the severity of OSAHS improved (37). In the process of CPAP therapy, the opposite results may be affected by the air leakage or counterflow (38–40). Therefore, we will explore the effects of surgical therapy for improving AHI on the ocular surface and MG in this study.

## Methods

This study was conducted in the Department of Ophthalmology and Otorhinolaryngology at the Second Xiangya Hospital of Central South University between August 2019 and April 2021. This study was conducted according to the Declaration of Helsinki and approved by the Ethics Committee of the Second Xiangya Hospital of Central South University (Number: 2021205).

### Participates

Patients who presented with snoring, nocturnal choking, and excessive daytime sleepiness underwent polysomnography (PSG) tests before their enrollment. According to the PSG results, a total of 103 patients (AHI ≥ 5) were included in our study. Based on the AHI, the severity of OSAHS was classified as follows: mild, 5/h ≤ AHI <15/h; moderate, 15/h ≤ AHI <30/h; severe, AHI ≥ 30/h (41). Twenty-one patients with mild OSAHS (Group A), 20 patients with moderate OSAHS (Group B) and 62 patients with severe OSAHS (Group C) were included in this study. Only the data of the right eyes were analyzed. Exclusion criteria included a history of ocular surgery and trauma, contact lens use, diabetes mellitus, glaucoma, thyroid-associated ophthalmopathy, chronic topical medication (anti-glaucoma eye drops), keratography, conjunctivitis, high myopia, rheumatic diseases and OSAHS patients receiving treatment. All subjects underwent detailed ophthalmological examinations. The examination was performed sequentially as follows: Keratograph 5M, visual acuity assessment, slit-lamp examination of the anterior segment, corneal fluorescein staining (CFS), ocular surface disease index (OSDI) questionnaire, Schirmer I test (SIT) and measurement of serum lipids. The results of PSG were blinded to the examiner during ophthalmic examination.

### Keratograph 5M

Keratograph 5M was applied to non-invasively measure certain parameters of the ocular surface, including tear meniscus height (TMH), non-invasive break-up time of tear film (NIBUT), lipid layer grading (LLG), bulbar redness index (BRI) and infrared meibography (42–44). The TMH was measured with an integrated ruler on the vertical line through the pupil center. The NIBUT was comprised of the first NIBUT (fNIBUT) and the average NIBUT (avNIBUT). The level of NIBUT was classified as follows: normal 0 (fNIBUT ≥ 10 s and avNIBUT ≥ 14 s); borderline 1 (between 0 and 2) or Dry eye (fNIBUT ≤ 5 s or avNIBUT ≤ 6 s) (45). Based on structural clarity and color richness, the lipid layer grading was divided into three levels: thin (level 1 with fizzy structure and gray color), normal (level 2 with clear structure and rich color), and thick (level 3 with extremely clear structure and rich color) (42). The BRI, comprising nasal limbal, temporal limbal, temporal bulbar, nasal bulbar and global regions, appears together with the total area analyzed (mm^2^), which is calculated by the equipment software as the area percentage ratio between the vessels and the rest of the analyzed area. The BRI ranges from 0.0 to 4.0, and the more blood vessels there are, the higher the score (46). Infrared images of the MG were taken after eyelid eversion, and the loss of the MG was calculated by Photoshop and Image J software as the area percentage ratio of dropout sections to the total area, including the upper and lower eyelids. Additionally, the distortion of ducts was defined as distortion of > 45° in at least one MG in the upper or lower eyelid (47).

### Corneal Fluorescein Staining

The CFS was performed by placing a wet sodium fluorescein test strip in the lower conjunctival sac, and observed the dye under the blue light from a slit-lamp. The CFS was ranged from 0 to 12, which was the sum of the scores of the four corneal quadrants (superior nasal, inferior nasal, superior temporal and inferior temporal) scored individually as 0 (no staining), 1 (mild staining with 1–30 dots of stains), 2 (moderate staining between 1 and 3), or 3 (severe staining with confluent stains, filaments or ulcers) (48).

### Ocular Surface Disease Index Questionnaire

The OSDI questionnaire is composed of three sections, involving eye symptoms, environmental factors and visual function. The score of each item on the questionnaire is 0 to 4. The OSDI score is the sum of the scores of 12 items, multiplied by 25, and then divided by the number of questions answered (49).

### Schirmer I Test

After the subjects accomplished the corneal staining, they were given a 30 min rest. The Schirmer I test strip was placed into the lower eyelid one-third of the distance from the lateral canthus and the wetting distance was recorded after 5 min.

### Diagnostic Criteria of LDDE, FES, and MGD

The diagnostical criteria of lipid-deficient dry eye (LDDE): LDDE is caused by the abnormal quality and quantity of the lipid layer, such as MGD, or various factors causing increased tear evaporation. The tear film stability decreased significantly (FBUT or NIBUT ≤ 5 s), and the tear secretion was slightly abnormal (10 mm/5 min ≤ SIT ≤ 12 mm/5 min), which tended to be considered as lipid-deficient dry eye (50). The floppy eyelid syndrome (FES) was defined as lid hyperlaxity, easily everted upper eyelids and reactive conjunctival changes (51). The diagnostic criteria of meibomian gland dysfunction (MGD) were abnormalities of the lid margin, meibomian gland orifices, meibum secretion and lipid layer thickness, dropout of the meibomian gland and ocular symptoms (52).

### Statistical Analysis

Statistical analysis was executed with software package SPSS version 25.0 (SPSS Inc., Chicago, IL, USA). Descriptive variables are expressed as the mean ± standard deviation (SD). The normality distribution of the data was tested by the Kolmogorov-Smirnov test. The variables with a non-normal distribution were analyzed by means of a non-parametric test. The variables among the three groups were analyzed through the non-parametric Kruskal-Wallis H-test. The non-parametric Mann–Whitney U test was applied to compare two groups. The enumeration data was analyzed using Chi-square tests, including sex and the incidence of DES, FES and MGD. The paired Student's *T*-test was applied to compare the preoperative and postoperative differences. Spearman's correlation was applied to analyze the relationship between the variables. *P*-value <0.05 was considered as statistically significant.

## Results

**Figure 1:**
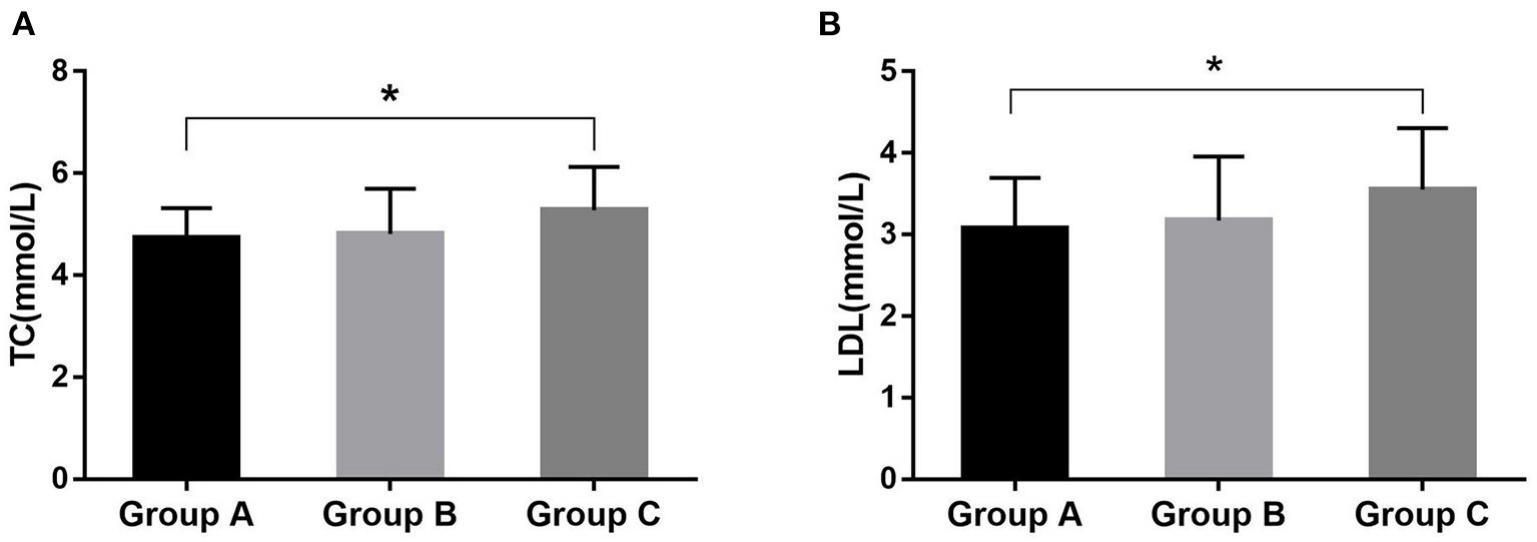
The significant difference in serum lipids among these groups. The difference was significant for TC **(A)** and LDL **(B)** between Groups A and C. *P < 0.05.

The general information of the subjects is shown in Table 1. The ratio of males to females was 96–7 and body mass index (BMI) was 28.0 ± 3.0 kg/m^2^ in severe OSAHS patients, which was significantly higher in Group C than in Group A (*p* < 0.001). Some patients were taking hypolipidemic drugs or had a lack of results, resulting in the partial loss of serum lipid data (Table 2). The total cholesterol (TC) values were 4.74 ± 0.58 mmol/L, 4.81 ± 0.88 mmol/L, and 5.28 ± 0.84 mmol/L for Groups A, B, and C, respectively. The low-density lipoprotein (LDL) values were 3.08 ± 0.62 mmol/L, 3.17 ± 0.78 mmol/L, and 3.55 ± 0.76 mmol/L for Groups A, B, and C, respectively. These values were significantly different between Groups A and C (*p* = 0.014 and *p* = 0.020) (Table 2; Figure 1). The high-density lipoprotein (HDL) or triglyceride (TG) levels was not significantly different among these groups.

**Table 1:** General information of the OSAHS patients.

	**Group A (*n =* 21)**	**Group B (*n =* 20)**	**Group C (*n =* 62)**
Age	35.6 ± 10.7	38.6 ± 7.3	40.1 ± 8.4
Sex (male/female)	17/4	18/2	61/1^*^
BMI (kg/m^2^)	25.2 ± 2.5	26.6 ± 3.1	28.0 ± 3.0^***^
AHI	9.8 ± 3.1	24.8 ± 4.4	57.2 ± 15.8^###^
mSaO_2_	82.1 ± 8.0	74.7 ± 7.8	63 ± 9.3^###^

*BMI, body mass index; AHI, apnea hypopnea index; mSaO_2_, minimum oxygen saturation*.

*Compared with Group A:*

* *P < 0.05*,

*** *P < 0.001, Mann–Whitney U test*.

### *P < 0.001, Kruskal-Wallis H-test*.

**Table 2:** The serum lipid levels of the OSAHS patients.

**Serum lipids**	**Group A (*n =* 19)**	**Group B (*n =* 15)**	**Group C (*n =* 48)**
TC (2.90–5.20)	4.74 ± 0.58	4.81 ± 0.88	5.28 ± 0.84^*^
TG (<1.71)	2.56 ± 2.15	2.34 ± 1.19	2.80 ± 1.61
HDL (>1.04)	1.10 ± 0.27	1.12 ± 0.21	1.06 ± 0.22
LDL (<3.12)	3.08 ± 0.62	3.17 ± 0.78	3.55 ± 0.76^*^

*TC, total cholesterol; TG, triglycerides; HDL, high density lipoprotein; LDL, low density lipoprotein. Compared with Group A:*

* *P < 0.05, Mann–Whitney U test*.

**Figure 2:**
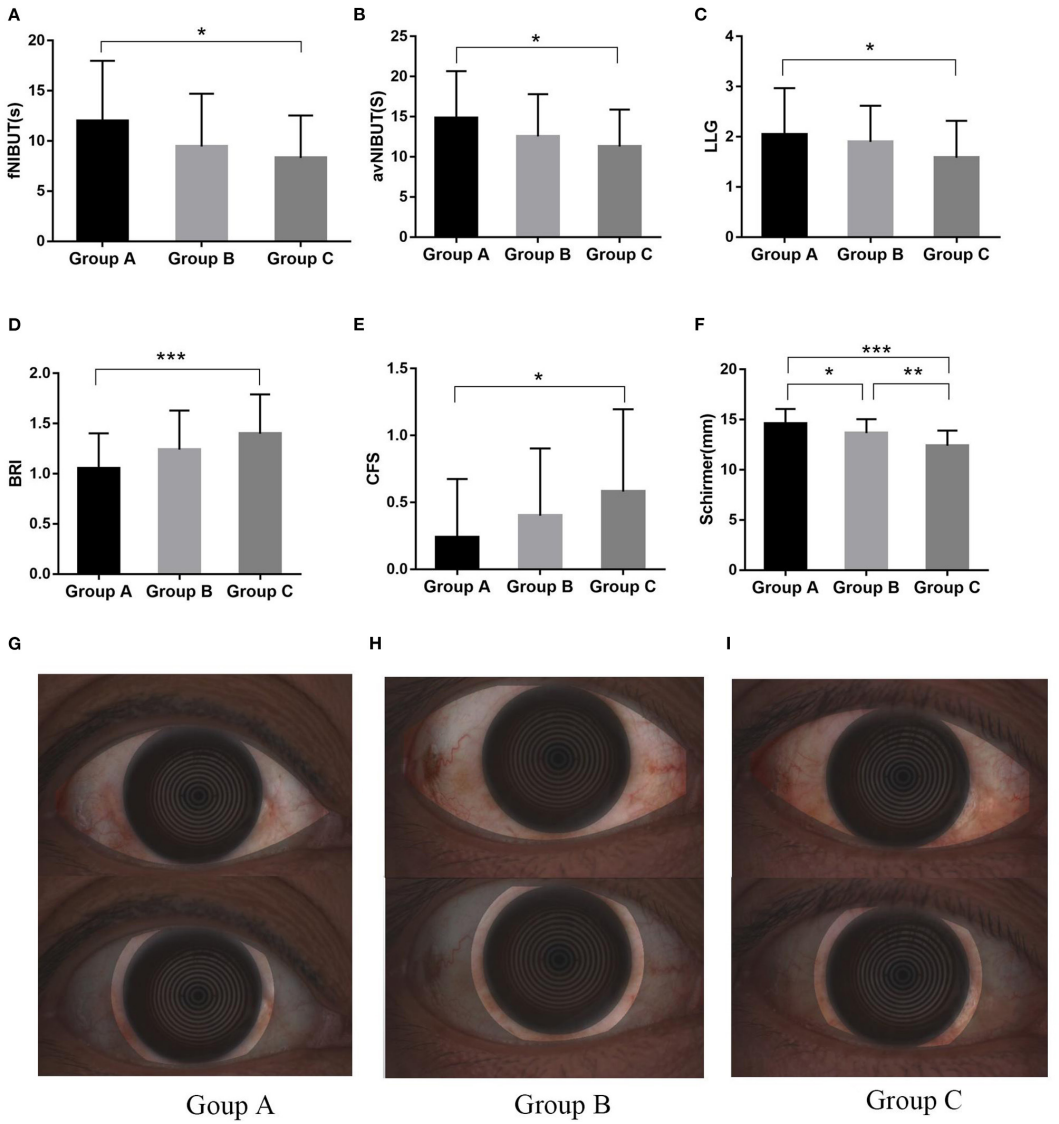
The significant difference in Ocular Surface Parameters and bulbar redness index among these groups. The fNIBUT **(A)**, avNIBUT **(B)**, and LLG **(C)** were lower in severe OSAHS patients. The difference was significant between Groups A and C for BRI **(D)**, as well as for CFS **(E)**. The Schirmer in the Group A was significantly higher than in Group B (*P* = 0.025) and in Group C **(F)**. The bulbar redness index in mild **(G)**, moderate **(H)**, and severe **(I)** OSAHS patients. *P < 0.05; **P < 0.01; ***P < 0.001.

The ocular surface parameters are presented in Table 3 and Figure 2. The OSDI score and TMH were not significant difference among the groups (*p* = 0.291 and *p* = 0.287, respectively). The fNIBUT and avNIBUT were significantly different between the group A and Group C. In Group C, fNIBUT and avNIBUT were shorter than in Group A (*p* = 0.015 and *p* = 0.018, respectively). Similarly, the BRI, LLG and CFS has significant difference among these groups. In Group A, both BRI and CFS were lower and LLG was higher than in Group C (*p* < 0.001, *p* = 0.022 and *p* = 0.036, respectively). The Schirmer values were 14.7 ± 1.5 mm, 13.7 ± 1.4 mm, and 12.4 ± 1.5 mm, respectively. The Schirmer in the Group A was significantly higher than in Group B (*p* = 0.025) and in Group C (*p* < 0.001).

**Table 3:** The ocular surface parameters of the OSAHS patients.

	**Group A (*n =* 21)**	**Group B (*n =* 20)**	**Group C (*n =* 62)**
OSDI score	3.4 ± 4.6	3.6 ± 4.3	5.2 ± 6.0
TMH (mm)	0.25 ± 0.06	0.24 ± 0.05	0.23 ± 0.05
fNIBUT (mm)	12.01 ± 5.98	12.55 ± 5.24	8.27 ± 4.18^*^
avNIBUT (mm)	14.94 ± 5.83	12.55 ± 5.24	11.52 ± 4.81^*^
BRI	1.0 ± 0.4	1.2 ± 0.4	1.4 ± 0.4^***^
LLG	2.1 ± 0.9	1.8 ± 0.7	1.6 ± 0.7^*^
Plugged orifices (*n*, %)	8 (38.10%)	11 (55%)	44 (70.97%)^**^
Distortion (*n*, %)	6 (28.57%)	9 (45%)	36 (58.06%)^*^
LRUE (%)	25.71 ± 12.50	30.40 ± 13.07	36.38 ± 18.08^*^
LRLE (%)	26.45 ± 16.54	26.87 ± 25.57	27.03 ± 23.22
CFS	0.2 ± 0.4	0.4 ± 0.5	0.6 ± 0.6^*^
Schirmer (mm)	14.7 ± 1.5^#^	13.7 ± 1.4^*^	12.4 ± 1.5^***^^##^

*OSDI, ocular surface disease index; TMH, tear meniscus height; fNIBUT, first non-invasive breakup time; avNIBUT, average non-invasive breakup time; BRI, bulbar redness index; LLG, lipid layer grading; LRUE, loss ratio of the upper eyelid; LRLE, loss ratio of the lower eyelid; CFS, corneal fluorescein staining; Compared with Group A:*

* *P < 0.05*,

** *P < 0.01*,

*** *P < 0.001, Mann–Whitney U test. Compared with Group B:*

# *P < 0.05*,

## *P < 0.01, Mann–Whitney U test*.

**Figure 3:**
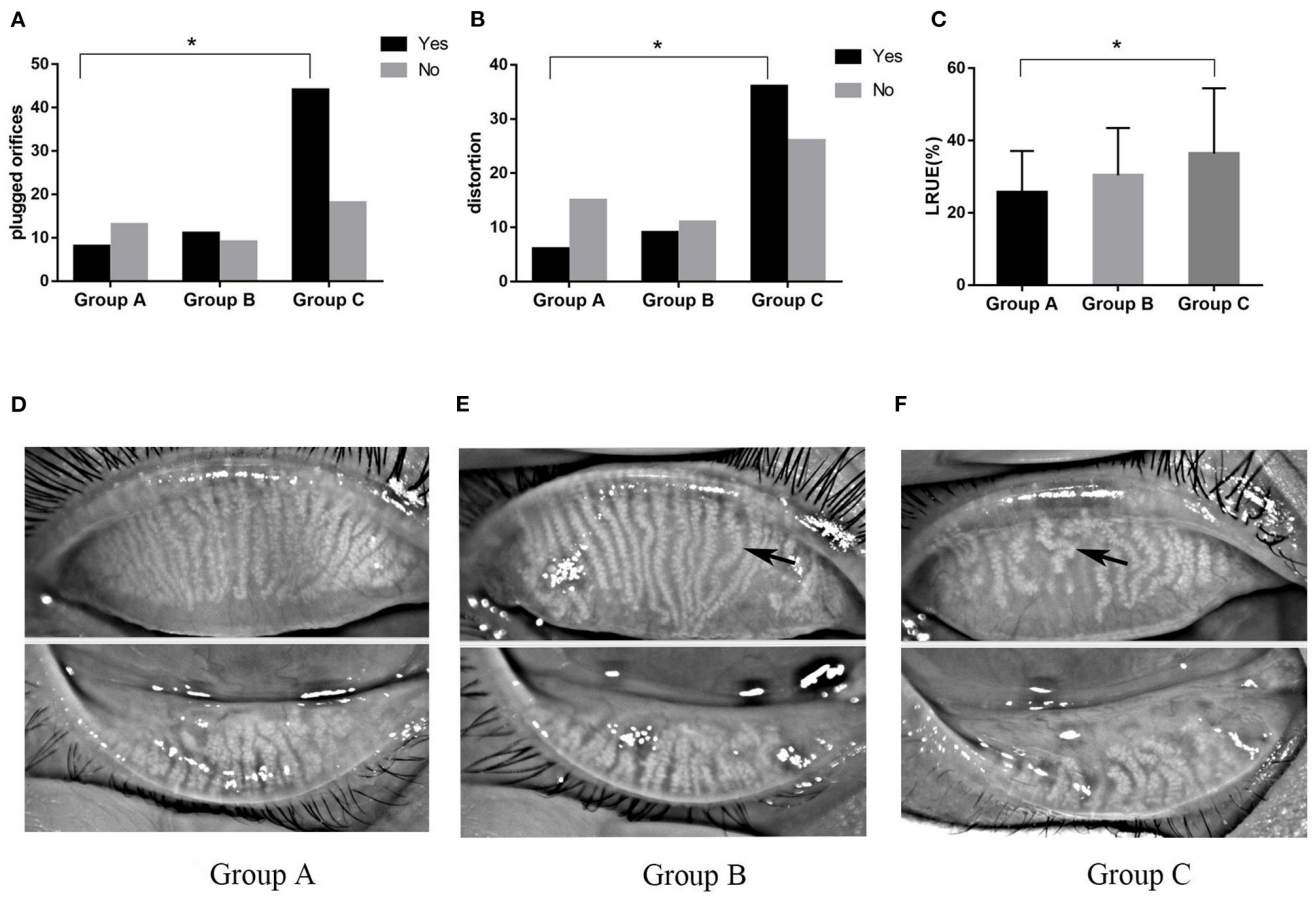
The morphological changes of MG. The number of plugged orifices **(A)** and distortion **(B)** of the MG was higher in severe OSAHS patients. The loss ratio of the upper eyelid **(C)** was significantly different between Groups A and C. The loss of meibomian gland in mild **(D)**, moderate **(E)**, and severe **(F)** OSAHS patients. Black arrows demonstrate the distortion in the duct. *P < 0.05.

The morphology of MG was significantly altered in severe OSAHS patients (Figure 3). Plugged orifices and distortion of ducts were observed in 70.97 and 58.06% of patients with severe OSAHS, respectively (*p* = 0.007, *p* = 0.019). In the Group C, the loss ratio of the upper eyelid was more severe than that in the Group A (*p* = 0.017). However, that of the lower eyelid was much higher but not significantly different. The incidence of DES and MGD was higher in Group C than in the Group A (*p* = 0.045 and *p* = 0.013, respectively), and all of dry eye was lipid-deficient dry eye (LDDE). The FES was observed more frequently in severe OSAHS patients (*p* = 0.029) (Table 4).

**Table 4:** The incidence of DES, FES and MGD in patients.

	**Group A (*n =* 21)**	**Group B (*n =* 20)**	**Group C (*n =* 62)**
DES (*n*, %)	4 (19.1%)	6(30%)	27 (43.6%)^*^
LDDE/ATD	4/0	6/0	27/0
FES (*n*, %)	0	0	12 (19.4%)^*^
MGD (*n*, %)	7 (33.3%)	9 (45%)	40 (64.5%)^*^

*DES, dry eye syndrome; LDDE, lipid-deficient dry eye; ATD, aqueous tear deficiency; FES, floppy eyelid syndrome; MGD, meibomian gland dysfunction. Compared with Group A:*

* *P < 0.05, Chi-square test*.

**Figure 4:**
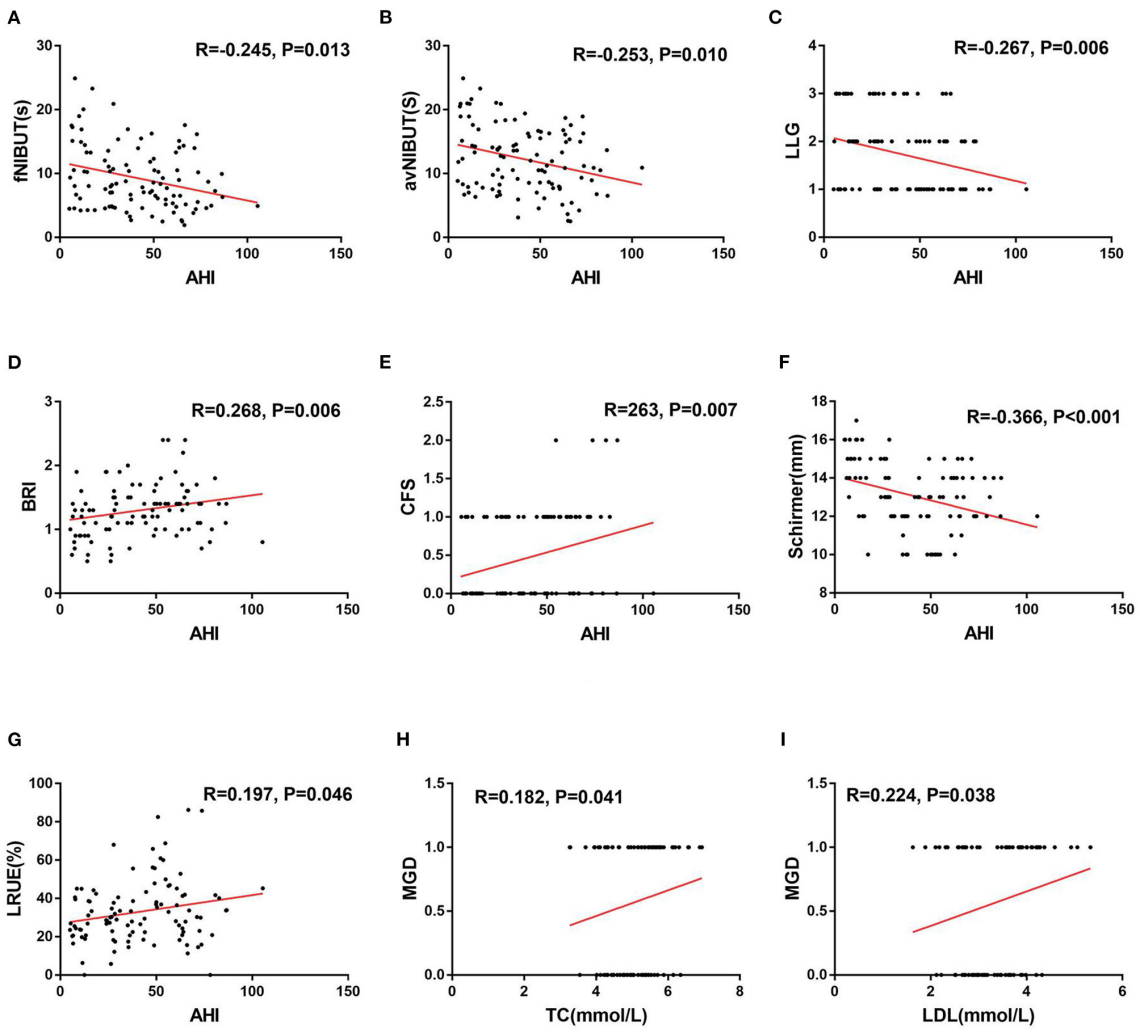
Correlations between the AHI and ocular surface parameters, MGD and serum lipids. The fNIBUT **(A)**, avNIBUT **(B)**, LLG **(C)**, and Schirmer **(F)** were significantly negatively correlated with the AHI. The BRI **(D)**, CFS **(E)**, and LRUE **(G)** were positively correlated with the AHI. The TC **(H)** and LDL**(I)** were positively correlated with MGD. R, Spearman's correlation coefficient, −1 ≤ R ≤ 1. A *P*-value < 0.05 was considered to be statistically significant.

The correlation analyses are described in detail in Tables 5, 6. The fNIBUT, avNIBUT, Schirmer values and LLG were significantly negatively correlated with the AHI (*R* = −0.263, *p* = 0.006; *R* = −0.200, *p* = 0.038; *R* = −0.200, *p* < 0.001; *R* = −0.211, *p* = 0.029, respectively). Moreover, the BRI, CFS and LRUE were found to be significantly positively correlated with the AHI (*R* = 0.284, *p* = 0.003; *R* = 0.256, *p* = 0.008; *R* = 0.176, *p* = 0.040). The TC and LDL levels were significantly correlated with MGD (*R* = 0.221, *p* = 0.041; *R* = 0.246, *p* = 0.023, respectively) (Figure 4).

**Table 5:** The correlation between ocular surface parameters and the apnea hypopnea index.

	***R***	***P***
OSDI score	0.103	0.156
TMH	0.102	0.305
fNIBUT	−0.245	0.013^**^
avNIBUT	−0.253	0.010^*^
BRI	0.268	0.006^**^
LLG	−0.267	0.006^**^
LRUE (%)	0.197	0.046^*^
LRLE (%)	0.062	0.534
CFS	0.263	0.007^**^
Schirmer I	−0.366	<0.001^***^

*OSDI, ocular surface disease index; TMH, tear meniscus height; fNIBUT, first non-invasive breakup time; avNIBUT, average non-invasive breakup time; LLG, lipid layer grading; BRI, bulbar redness index; LRUE, loss ratio of the upper eyelid; LRLE, loss ratio of the lower eyelid; CFS, corneal fluorescein staining; R, Spearman's correlation coefficient, −1 ≤ R ≤ 1; P: Spearman's correlation analysis significance value*.

* *P-value < 0.05;*

** *P-value < 0.01;*

*** *P-value <0.001*.

**Table 6:** The correlation between MGD and serum lipids.

	***R***	***P***
TC	0.182	0.041^*^
TG	0.092	0.399
HDL	−0.165	0.128
LDL	0.224	0.038^*^

*TC, total cholesterol; TG, triglycerides; LDL, low density lipoprotein; HDL, high density lipoprotein. R, Spearman's correlation analysis coefficient, −1 ≤ R ≤ 1, P: Spearman's correlation analysis significance value*.

* *P value <0.05*.

A total of 103 OSAHS patients were enrolled in our study, of these patients, 66 cases were diagnosed with DES. 5 cases who underwent uvulopalatopharyngoplasty for OSAHS were followed up in 3 months, of which 3 cases were moderate OSAHS patients and 2 cases were severe OSAHS patients. The characteristics of the postoperative ocular surface are shown in Table 7. The AHI values were significantly different from the preoperative values, but no significant differences were found among other ocular surface characteristics.

**Table 7:** The ocular surface parameters of Pre- and Post-operation in patients.

	**Pre-operation (*n =* 5)**	**Post-operation (*n =* 5)**	***P-*value**
AHI	40.48 ± 25.38	16.42 ± 9.19	0.030^*^
OSDI score	2.27 ± 2.88	1.52 ± 1.86	0.363
TMH (mm)	0.25 ± 0.07	0.25 ± 0.04	0.932
fNIBUT (s)	7.49 ± 3.42	7.95 ± 6.28	0.905
avNIBUT (s)	11.17 ± 6.22	11.28 ± 6.78	0.977
LLG	1.8 ± 0.8	1.8 ± 0.8	-
BRI	1.58 ± 0.26	1.35 ± 0.32	0.319
LRUE (%)	34.22 ± 25.80	32.72 ± 24.65	0.316
LRLE (%)	24.43 ± 33.33	23.45 ± 32.98	0.294
CFS	0.83 ± 0.98	0.67 ± 0.82	0.363
Schirmer I (mm)	12.83 ± 2.82	13.33 ± 1.86	0.580

*AHI, apnea hypopnea index; TMH, tear meniscus height; fNIBUT, first non-invasive breakup time; avNIBUT, average non-invasive breakup time; LLG, lipid layer grading; BRI, bulbar redness index; LRUE, loss ratio of the upper eyelid; LRLE, loss ratio of the lower eyelid; CFS, corneal fluorescein staining*.

* *P-value < 0.05, paired Student's T-test*.

## Discussion

To our knowledge, this is the first study to evaluate certain parameters of the ocular surface for OSAHS patients by a non-invasive instrument, and to analyze the correlation between the ocular surface and the severity of OSAHS in terms of AHI. In our study, we found that the patients with severe OSAHS had higher BRI and CFS scores, which were positively correlated with the AHI. The BRI may be a potential objective index to estimate the severity of OSAHS in the clinic. In OSAHS patients, long-term chronic intermittent hypoxia has been found to increase oxidative stress, resulting in an upregulation of stress signaling cascades, such as HIF-1, NF-KB and Nrf2 (53). These pathways further incite a systematic chronic inflammatory response and immunological alterations (54), and increase the expression of proinflammatory cytokines and adhesion molecules, including IL-6, IL-8, TNFα, and Fas recptor-positive lymphocytes (55). Dilated conjunctival vessels and damaged epithelial cells release cytokines, resulting in a persistent inflammatory state that ultimately causes the apoptosis of goblet cells and keratoconjunctival epithelial cells (56). Therefore, inflammation may contribute to ocular surface damage in OSAHS patients. Local anti-inflammatory treatments may improve the ocular surface damage caused by OSAHS, but this needs further research.

Additionally, the loss ratio of the upper eyelid MG was higher in severe OSAHS patients, and the incidence of gland distortion, FES and MGD were positively correlated with the AHI. The proportion of OSAHS patients with FES varies from 2 to 40%, and the occurrence of FES in OSAHS patients indicates that the disease severity is more serious (57). This may be related to the increase in elastase activity in eyelid connective tissue caused by longstanding hypoxia, resulting in the degradation and fracture of eyelid elastic fibers (18, 57). We hypothesize that the reason for the above change in OSAHS patients may be due to the long-term intermittent hypoxia that leads to an increase in matrix metalloproteinase activity in eyelid connective tissue, the degradation of elastic fibers, a decrease in eyelid tension, and the weakening of the squeeze-driving effect of the orbicularis oculi muscle on the MG, resulting in stasis of the meibum. With the accumulation of meibum, the pressure in the conduct gradually increases, which contributes to the atrophy and dropout of the MG eventually. An additional reason could be that intermittent hypoxia increases the degradation of the MG matrix, causing the gland duct to be distorted and irregular. This could result in the increased resistance to meibum secretion, causing the stasis of meibum. This may be followed by its accumulation and degeneration, which would change the quantity and quality of meibum secretion.

Furthermore, the plugged orifices increase the intraluminal pressure, forming a vicious circle that leads to atrophy and dropout of the MG. Finally, another reason could be due to the lower androgen level in OSAHS patients than that in normal people (58). The MG contains the androgen receptor, which can improve the quality of meibum secretion (59). Therefore, the decreased androgen level may be one of the reasons for the abnormal morphology and function of the MG.

In 2019, Karaca et al. observed dropout and morphological changes in the MG in OSAHS patients with a slit-lamp biomicroscope (19). They first reported that compared with mild OSAHS patients, severe OSAHS patients had a higher degree of upper meiboscore and morphological alterations, such as thinning, dilatation and distortion. However, the lower meiboscore was not significantly different. Similarly, our findings were in accordance with their results. Muhafiz et al. found that the loss of the MG in both the upper and lower eyelid was more severe in the OSAHS group than that in the control group (22). Research has found that the loss of MG was significantly different in the upper eyelid and lower eyelid between Sjögren's syndrome and non-Sjögren's syndrome (45). Heiko and Youngsub et al. observed that the loss of MG in the lower eyelid was more severe than that in the upper eyelid in MGD patients (60, 61). This phenomenon can be explained by the evident action of orbicularis oculi muscles in the upper eyelid during blinking. In addition, the direction of meibum delivery of the upper eyelid is consistent with the direction of gravity, while that of the lower eyelid is the opposite. Therefore, the meibum from the lower eyelid is more easily stagnate in the orifices, leading to duct dilation, acinar atrophy and eventual loss of MG. In OSAHS patients, the pathogenesis of FES is mainly related to the elastin located in the tarsal plate of the upper eyelid (7), and long-term hypoxia decreases the amount of elastin, causing prominent upper eyelid laxity (62, 63). This may result in a relative deterioration in easy and persistent secretion of meibum from the upper eyelid. Thus, as shown in this study, the loss of the MG in the upper eyelid appears to be more prominent.

The tear film has a complex structure, and it is followed by a lipid layer, aqueous layer, and mucin layer from the outside to inside. The normal function of various tear functional units is vital to a stable tear film and heathy ocular surface (64). The MG is a type of sebaceous gland with tubule acinar structure and holocrine function. MG secrete meibum to the ocular surface, where it coats the aqueous layer and prevents tear evaporation and provides tear stability (65). Studies have shown that TBUT and Schirmer values were lower in OSAHS patients, and much more in moderate and severe OSAHS (18, 19, 21). Similarly, we also found these results in moderate and severe OSAHS, and they were associated with increasing OSAHS severity. Previous researches have found that the values of NIBUT were higher than FBUT (31, 66, 67). The reason could be that the fluorescein affects the stability of tear film, and has low Reproducibility (64), while Keratograph 5M is considered as a non-invasive and objective method that measure BUT by the analysis of disruption of the reflection of Placido rings onto the tear film, with good repeatability and reliability (29). Additionally, the lipid layer was thinner and the incidence of DES was higher in severe OSAHS patients, and the DES was lipid-deficient dry eye caused by MGD. As mentioned above, in patients with OSAHS, long-standing intermittent hypoxia causes systematic inflammation, resulting in distortion and dropout of the MG and subsequent MGD. MGD affects the quality of meibum, causing accelerated tear evaporation and decreased stability of tear film and tear hyperosmotic pressure. These conditions lead to ocular surface damage through a series of inflammatory cascade responses and are the main causes of lipid-deficient dry eye.

Studies have shown that elevations in TC, TG and LDL, and decreases in HDL in patients with OSAHS, and those alterations may be exacerbated with the increasing of OSAHS severity (68, 69). A possible reason is that long-standing intermittent hypoxia stimulates the production of stearoyl-coenzyme A desaturase-1 and reactive oxygen species. Increase production of these cause lipid peroxidation and sympathetic system dysfunction, resulting in disorders of lipid metabolism and dyslipidemia (69). In this study, TC and LDL levels were higher in severe OSAHS patients than in mild OSAHS patients. Meibum is mainly composed of wax and neutral sterol esters, with low amounts of polar lipids, triglycerides, and cholesterol, which have been reported to range from 1 to 2% (70). Alterations in the quality of meibum and plugged ducts are considered the core of MGD. Research has found that high levels of TG, TC and LDL are associated with MGD (71, 72). The melting point of normal meibum ranges from 30 to 34°C, but the melting point of cholesterol is 148°C because of structural differences (73). Therefore, meibum would have a higher melting point and be more viscous due to a higher concentration of cholesterol, thus plugging the ducts and resulting in MGD.

Currently, CPAP and surgery are the two major treatment modalities for OSAHS. CPAP is considered as the first-ling treatment for OSAHS, but patient compliance has been notably poor result from mask discomfort, nasal dryness and congestion, and difficulty adopting to the pressure, which decrease the usage and efficacy (74, 75). In the literature, there are 3 prospective studies on ocular surface problems in patients with OSAHS after CPAP therapy. Kadyan et al. and Acar et al. have reported that TBUT and ocular irritative symptoms were improved in OSAHS patients after CPAP therapy (35, 36). This may be explained by reduced displacement of mask due to OSAHS patients were in a supine position during CPAP. The blood oxygen levels were increased after long-term treatment, and the mechanical injury to the eyelid was reduced accordingly, thereby improving the ocular surface health. Hayiric et al. has demonstrated that the TBUT was decreased only in the right eyes between pre-CPAP and post-CPAP (37). They speculated that the inconsistency between the left and right eyes may be caused by the sleep posture. When in the left-lying position, air leakage promotes tear evaporation due to the shift of the CPAP mask, resulting in decreased TBUT and increased ocular irritative symptoms. The evaluation of the effects of CPAP therapy for improving AHI on the ocular surface has been influenced by a variety of factors, the following conclusions were controversial. Therefore, we sought to explore the changes of the ocular surface parameters in OSAHS patients after surgical treatment. In the present study, the OSAHS severity was significantly improved 3 months after surgery, but the ocular surface characteristics were not significantly different. The ocular surface damage in OSAHS patients cannot be reversed in a short time by improving the severity of OSAHS. However, there are some limitations in demonstrating the effects of surgery on ocular surface parameters due to small samples and short period of follow-up. It may be necessary to follow up with more cases and longer treatment cycles to observe the changes in ocular surface parameters to explain whether improving the severity of OSAHS by surgery can help to improve the ocular surface condition. This could thereby achieve the purpose of clinically intervening in the progression of dry eye in OSAHS patients or treating dry eye in OSAHS patients.

## Data Availability Statement

The raw data supporting the conclusions of this article will be made available by the authors, without undue reservation.

## Ethics Statement

The studies involving human participants were reviewed and approved by the Ethics Committee of the Second Xiangya Hospital of Central South University. The patients/participants provided their written informed consent to participate in this study.

## Author Contributions

SLiu and SLi: analysis and interpretation of data and drafting and revising the article. ML and SZ: participate in survey and data collection. LZ and BC: substantial contributions to conception and design and revising it critically for important intellectual content. All authors approved the final manuscript.

## Funding

This work was supported by Natural Science Foundation of Hunan Province (No. 2020JJ5845).

## Conflict of Interest

The authors declare that the research was conducted in the absence of any commercial or financial relationships that could be construed as a potential conflict of interest.

## Publisher's Note

All claims expressed in this article are solely those of the authors and do not necessarily represent those of their affiliated organizations, or those of the publisher, the editors and the reviewers. Any product that may be evaluated in this article, or claim that may be made by its manufacturer, is not guaranteed or endorsed by the publisher.
